# Integrating omics to characterize eco‐physiological adaptations: How moose diet and metabolism differ across biogeographic zones

**DOI:** 10.1002/ece3.7265

**Published:** 2021-03-04

**Authors:** Christian Fohringer, Ilona Dudka, Robert Spitzer, Fredrik Stenbacka, Olena Rzhepishevska, Joris P. G. M. Cromsigt, Gerhard Gröbner, Göran Ericsson, Navinder J. Singh

**Affiliations:** ^1^ Department of Wildlife, Fish, and Environmental Studies Swedish University of Agricultural Sciences Umeå Sweden; ^2^ Department of Chemistry Umeå University Umeå Sweden

**Keywords:** biomarker, DNA‐metabarcoding, energy metabolism, metabolomics, nutritional ecology, starvation, ungulate

## Abstract

1. With accelerated land conversion and global heating at northern latitudes, it becomes crucial to understand, how life histories of animals in extreme environments adapt to these changes. Animals may either adapt by adjusting foraging behavior or through physiological responses, including adjusting their energy metabolism or both. Until now, it has been difficult to study such adaptations in free‐ranging animals due to methodological constraints that prevent extensive spatiotemporal coverage of ecological and physiological data.
2. Through a novel approach of combining DNA‐metabarcoding and nuclear magnetic resonance (NMR)‐based metabolomics, we aim to elucidate the links between diets and metabolism in Scandinavian moose *Alces alces* over three biogeographic zones using a unique dataset of 265 marked individuals.
3. Based on 17 diet items, we identified four different classes of diet types that match browse species availability in respective ecoregions in northern Sweden. Individuals in the boreal zone consumed predominantly pine and had the least diverse diets, while individuals with highest diet diversity occurred in the coastal areas. Males exhibited lower average diet diversity than females.
4. We identified several molecular markers indicating metabolic constraints linked to diet constraints in terms of food availability during winter. While animals consuming pine had higher lipid, phospocholine, and glycerophosphocholine concentrations in their serum than other diet types, birch‐ and willow/aspen‐rich diets exhibit elevated concentrations of several amino acids. The individuals with highest diet diversity had increased levels of ketone bodies, indicating extensive periods of starvation for these individuals.
5. Our results show how the adaptive capacity of moose at the eco‐physiological level varies over a large eco‐geographic scale and how it responds to land use pressures. In light of extensive ongoing climate and land use changes, these findings pave the way for future scenario building for animal adaptive capacity.

## Graphical abstract

With accelerated land conversion and global heating at northern latitudes, it becomes crucial to understand, how life histories of animals in extreme environments adapt to these changes. By integrating omics, we characterize eco‐physiological adaptations that show how moose diet and metabolism differ across three biogeographic zones. The biomarkers identified in this study pave the way for future scenario building for animal adaptive capacity.

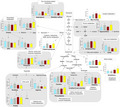

## 1 INTRODUCTION

Global heating and exploitation of natural resources are affecting ecosystems globally with their impacts particularly accelerated and elevated at high latitudes (Berglöv et al., 2015; Post et al., 2019). However, we still know relatively little about how animals in northern ecosystems are adapted to these latitudes and how they may adapt to changes in future. Spatio‐temporal adaptations of animals to temperatures, snow coverage, food type, and food availability may provide clues to how these organisms would react to climate shifts and other environmental changes caused by human activity (Bronson, 2009; Neumann et al., 2020; Sheriff et al., 2013).

In highly seasonal environments, animals may adapt to seasonal constraints by reducing metabolic expenditure in combination with foraging and starvation bouts when food is limited (McCue, 2010). Starvation and adaptation are reflected at a biochemical level that can be assessed by measuring metabolites in body fluids (Pagano et al., 2018). Hence, metabolic profiles can be explored to (a) understand the relationships between physiological responses and environmental factors and (b) to identify specific biomarkers (e.g., naturally occurring molecules) of distinct environmental responses, and (c) monitor the uptake of trophic biomarkers in individual organisms. These biomarkers can be identified and evaluated on the scale of entire populations or ecosystems (Galloway & Budge, 2020).

In recent years, metabolomics has emerged as a powerful approach to understand on a molecular level the organismal response to environmental stress and to identify specific biomarkers for distinct pathologies or physiological responses to environmental change (Chiu et al., 2017). Nuclear magnetic resonance (NMR)‐based metabolomics has become an ideal method for such studies due to its undiscriminating character which allows identification and quantification of all major metabolites, ease of sample preparation, unmatched cross‐laboratory reproducibility, and lack of sampling bias (Beckonert et al., 2007). NMR is ideal to generate hypotheses involving complex environmental stressors for which the mode of actions is still unknown (Lankadurai et al., 2013). The potential of environmental metabolomics on wild free‐ranging vertebrates to identify biomarkers based on demographic variables has previously been demonstrated in Mongolian gerbils *Meriones unguiculatus* and American black bears *Ursus americanus*, respectively (Niemuth & Stoskopf, 2014; Shi et al., 2015). The vast majority of animal species still remains unstudied.

Metabolic demands and environmental constraints also lead to variation in individual foraging patterns, which can be assessed in their diets. DNA‐based analysis of fecal samples using metabarcoding is another powerful omics approach to understand fine‐scale foraging behavior (Kartzinel et al., 2015; Kowalczyk et al., 2019; Pansu et al., 2019). A distinct advantage of the DNA‐metabarcoding approach is its independence from observer bias, accurate identification of species and efficiency when handling large sample sizes. This makes this approach ideal for studying fine‐scale individual variation in diets across large spatial and temporal scales.

The coupling of the two omics approaches (DNA‐metabarcoding and NMR‐based metabolomics) covering both high taxonomic diversity as well as high functional diversity is therefore a promising way to determine the degree at which diet and metabolic responses influence each other and to what extent they vary across individual animals (Taberlet et al., 2018). These linkages may provide even further important insights on the consequences of foraging and physiological adaptations on animal demography and behavior and ultimately their population persistence.

Moose *Alces alces* is a highly relevant model species to understand the physiological adaptations of an animal species to environmental gradients. Moose are widely distributed across the northern hemisphere and therefore encounter a variety of eco‐geographic zones characterized by differences in climatic conditions, topography as well as forage species (Neumann et al., 2020; Singh et al., 2012; Spitzer, 2019). They experience food limitation during winter due to extensive snow cover (Parikh et al., 2017; Shipley et al., 1998) and hence utilize built up body stores (Moen et al., 1997; Parker et al., 2009) as well as reduce activity and body temperature (Allen, Dorey, et al., 2016; Græsli et al., 2020). The energy metabolism of moose is likely influenced by forage availability and local habitat composition (Felton et al., 2020; Spitzer, 2019). However, little is known about the consequences of variation in diets on moose physiology, and the energetic costs and benefits associated with different diet types.

The main objective of this study is to characterize the physiological and dietary adaptations of moose across a biogeographic gradient. This crucial bit of knowledge will pave the way toward predictions on the future survival and adaptive capacity of moose and similar, widely distributed, and generalist species. This is especially important in light of climate and land use changes, such as resource extraction, that are occurring across the globe.

## 2 MATERIAL AND METHODS

### 2.1 Study area

**Figure 1:**
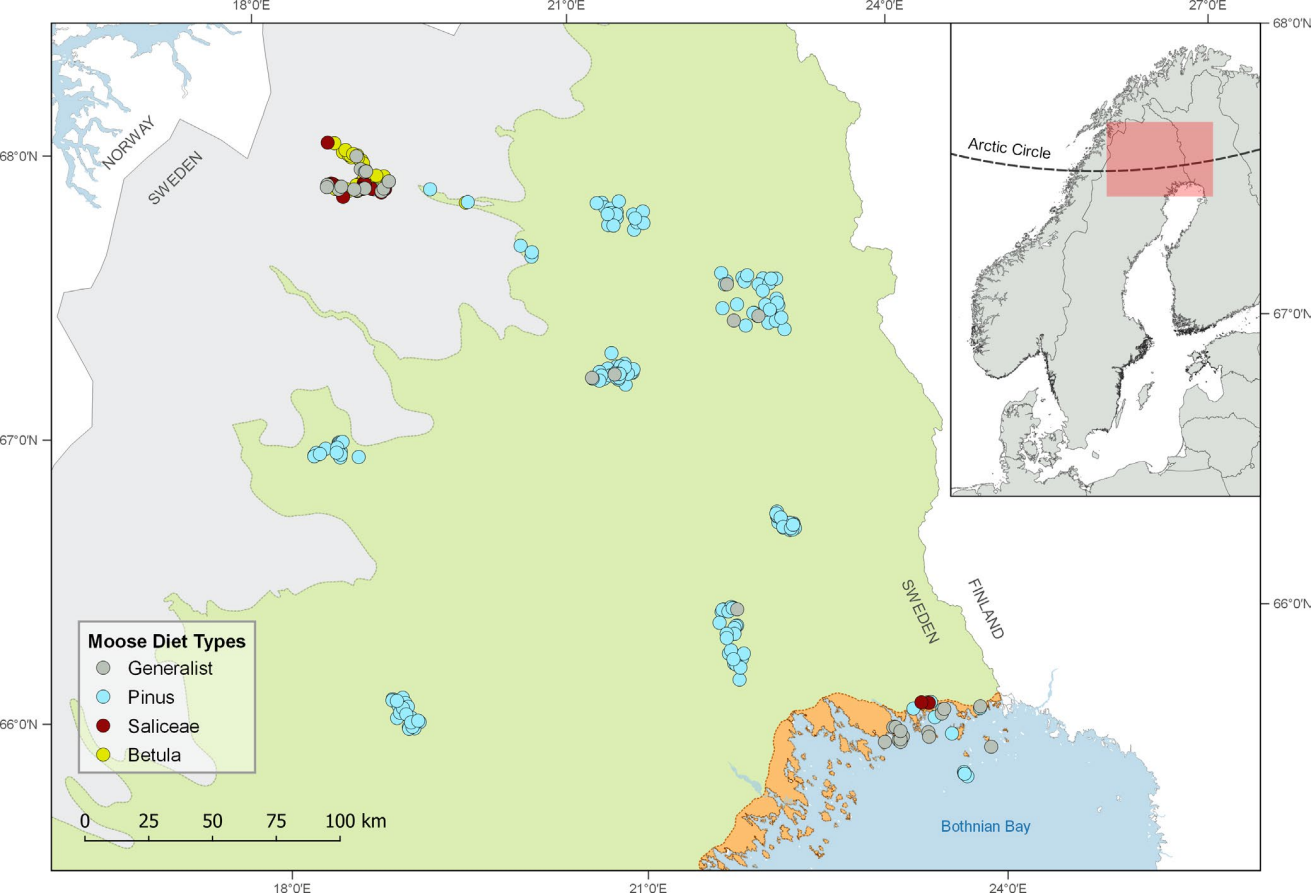
Moose winter diet types per capture location of 264 moose captured across all major ecoregions (gray—montane, green—boreal, orange—coastal) in northern Sweden. Diet types are categorized based on individuals consuming more (“specialists”: *Betula*, *Pinus*, and Saliceae) or less (“generalist”) than 60% of a single diet item

The study area covers a gradient across the Swedish sub‐Arctic biome (Figure 1), between 18.4–24.0ºE and 65.6–68.0ºN, and spanning altitudes from 3 to 674 m a.s.l. The study area is demarcated into “montane” (tundra) and “boreal” (taiga) based on Ecoregions 2017 (Dinerstein et al., 2017). Moose was captured in these two ecoregions and in an additional area around the archipelago of the Bothnian Bay that was classified as “coastal.”

**Figure A1:**
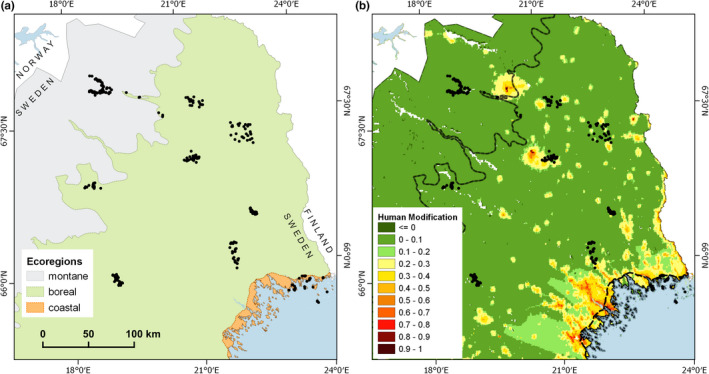
Maps representing the study area. A) The three ecoregions where moose were sampled (black dots). B) Map showing the human modification of the landscape (Source: Kennedy et al., 2019). The green areas are claimed as unmodified lands, representing boreal forests and the less productive and remote areas in high latitudes, inaccessible permanent rock and ice, or within tundra, and to lesser extent montane grasslands. The areas are claimed to have a low degree of human modification (0 < HMc ≤0.1), and largely reside ≥ 10 km away from more modified edges. Nevertheless, the yellow to red gradient demonstrates high modifications. In our study area, these represent mostly settlements and mines

While mining, hydro‐ and wind‐power plants, tourism and military activities are among the dominating land use factors, the spatially most extensive extractive land use in the study area is forestry (Fohringer et al., in review). Scots pine (*Pinus sylvestris*) is the dominant commercially important tree species (Swedish Forest Agency, 2010) and a predominant winter forage for moose across northern Fennoscandia, especially when growing in young plantation stands (Bergqvist et al., 2018; Milligan & Koricheva, 2013). No forestry is carried out in the montane ecoregion that is characterized by mountain birch *Betula pubescens* as the dominating tree species. Moreover, most animals in the coastal ecoregion were captured in the archipelago that is partly located in a national park (Haparanda Skärgård) where overall little forest management occurs. The islands in the archipelago offer a mosaic of different forest types, with Scots pine and Norway spruce *Picea abies* still being the dominating tree species, but generally more open habitat compared to the mainland. Otherwise, all captures were carried out in areas in production/managed forest, dominated by Scots pine and Norway spruce. Generally, land use in our study area intensifies from the montane to the coastal habitat (see Appendix, Figure A1).

### 2.2 Data collection and sampling

This study includes 265 free‐ranging adult moose captured between the winters of 2008 and 2017 (between February 14 and March 31), and following a standard monitoring procedure. Animals were immobilized from helicopter via dart injection (Kreeger & Arnemo, 2018) with a CO_2_‐powered rifle (Dan‐Inject) with the drug combination of 4.5 mg etorphine (Captivon® 98 Etorphine HCl 9.8 mg/ml, Wildlife Pharmaceuticals (PTY) Ltd.) and 50 mg xylazine (Xylased® 500 mg, Bioveta, a.s.) (Evans et al., 2012; Græsli et al., 2020; Lian et al., 2014). Fecal pellets were collected from the rectum into 50 ml screw cap tubes that were frozen within 6 hr and stored subsequently at −20ºC. For serum collection, whole blood was drawn from the jugular vein into 9 ml S‐Monovette® Z‐Gel collection tubes, processed according to the manufacturer's instructions and stored at −20°C. All moose captures are in line with the following proposed ethical permits: A124‐05, A116‐09, A50‐12, and A14‐15, granted by the Swedish Animal Ethics Committee.

GPS positions of capture locations and demographic data (sex, pregnancy status, number of calves) were collected during capture and this data was then stored in the Wireless Remote Animal Monitoring database (Dettki et al., 2014).

To assess moose winter diet across different landscapes, we identified the plant contents of fecal pellets and metabolites from corresponding serum samples from our study individuals from multiple capture areas across northern Sweden (Figure 1). Animals captured across northern Sweden were shown to be genetically similar (Blåhed et al., 2018; Niedzialkowska et al., 2016; Wennerstrom et al., 2016), and genetic admixture is possible due to their high propensity for seasonal migration (Allen, Dorey, et al., 2016; Singh et al., 2012). We focused on the winter period as this is when dietary constraints are highest and this is when the samples were taken. Snow cover lasts typically from 25 October to 5 May in the coastal habitat and 1 October to 25 May in the montane habitat (http://www.smhi.se/data/meteorologi/sno, last accessed 06/10/2020).

### 2.3 Dietary profiles

DNA from fecal samples was extracted following Spitzer (2019) and purification was carried out on a QIASymphony SP platform using the DSP DNA minikit (Quiagen) according to the manufacturer's instructions. To determine the diet composition, we used the universal primer pair Sper01_F & Sper01_R (Tab erlet et al., 2018) to amplify the P6‐loop of the *trnL* intron of chloroplasts. This commonly used metabarcoding marker for plants (e.g., De Barba et al. (2014), Tab erlet et al. (2012), Valentini et al. (2009)) has been well‐established for the study of herbivore diets (Kartzinel et al., 2015; Nichols et al., 2016; Pansu et al., 2019). For each fecal sample, PCR reactions were performed using technical triplicates. All experiments included extraction controls, PCR negative and positive controls, and PCR blanks. PCR products were purified using the MinElute PCR purification kit and sequenced on an Illumina HiSeq 2,500 platform using a paired‐end approach (2 x 125 bp). Sequence data were then processed using the OBITools software (Boyer et al., 2016) for (a) assembly and dereplication of reads, (b) matching sequences to samples, (c) denoising the data by removing singletons, low‐quality sequences, putative PCR and sequencing artifacts, and (d) taxonomic assignation of the remaining sequences. For the latter, we built a reference library for the local plant species by extracting the relevant parts of the EMBL (European Molecular Biology Laboratory) nucleotide database, the NCBI (National Center for Biotechnology Information) taxonomy, and a database for arcto‐boreal plant species and bryophytes (Soininen et al., 2015; Sønstebø et al., 2010; Willerslev et al., 2014). For further data processing, we used R (R Core Team, 2017). To facilitate data analysis at the ecological level, the final dataset was stored in a relational database using PostgreSQL (https://www.postgresql.org). Sequences without a match to a reference sequence and outlying PCR replicates were excluded from further analyses. We retained annotated sequences as molecular operational taxonomic units (MOTUs) and averaged the number of reads for each MOTU across the remaining PCR replicates for each sample. To confer the same weight to each fecal sample, read abundances were converted into relative read abundances (RRA), representing the proportion of each MOTU in each fecal sample. MOTUs that did not represent at least 2.5% in at least one fecal sample were removed from the final dataset (Bison et al., 2015). RRA is increasingly used as a quantitative measure for diet composition (Craine et al., 2015; Deagle et al., 2019; Kowalczyk et al., 2019; Pansu et al., 2019) and has been shown to yield similar conclusions to those derived from presence/absence data (Kartzinel et al., 2015; Kowalczyk et al., 2019; Willerslev et al., 2014). Because the taxonomic resolution of the trnL P6 barcode varies among plant families (Taberlet et al., 2007), sequences could frequently only be assigned at genus level or higher.

To determine diet types, we adopted the approach used by Shipley (2010) who defined moose consuming a “specialist” diet if >60% of the diet consisted of a single plant genus; conversely, a “generalist” moose diet was defined by no plant genus contributing >60% to the diet. Based on the diet composition, we quantified diet diversity as the Shannon entropy (Shannon–Wiener index) using the R package *vegan* (Oksanen et al., 2017). All statistical tests were carried out at a significance level of alpha =0.05.

### 2.4 Metabolomic profiles

Prior to analysis, serum samples were thawed and 300 μl were mixed with 300 μl 1.5 M deuterated phosphate buffer (NaH_2_PO_4_ and K_2_HPO_4_, including 0.1% TSP, pH 7.47) and transferred into 96‐well plates for NMR spectroscopy using a Gilson robot. Quality control samples were prepared by pooling all samples to monitor analytical variability of the metabolic profiling platform. The ^1^H NMR spectra were acquired using a Bruker 600 MHz AVANCE III spectrometer equipped with a 5 mm BBO broadband (1H/19F/2D) z‐gradient cryo‐probe at 311.0 K and an automatic temperature‐controlled high‐throughput sample changer (SampleJet, Bruker). One‐dimensional (1D) spectra were recorded using Carr‐Purcell‐Meiboom‐Gill (CPMG) sequence with water suppression in order to have enhanced visualization of low molecular weight compounds. The spectrum was acquired with a recycle delay of 4 s, 12‐kHz spectral width, 73,728 data points, 30 ms total spin‐echo time, total 64 scans, and 4 dummy scans. The acquired NMR spectra were manually corrected for the phase and the baseline with TopSpin 2.1 (Bruker Biospin).

### 2.5 Spectra analysis

^1^H NMR spectra were aligned using icoshift 1.2 and manual integration of peaks was performed to a linear baseline on all spectra in parallel using an in‐house developed Matlab routine as was applied before (Dudka et al., 2020; Virel et al., 2019). The integrated data from serum were normalized to the total sum of the spectrum to give the same total integration value for each spectrum. Identification of the metabolites was achieved by assigning their specific resonances according to the chemical shift values and multiplicities using the Chenomx NMR suite professional (version 7.72, Chenomx, Inc.) and the Human Metabolome Database (Wishart et al., 2018).

### 2.6 Univariate and multivariate analyses

Normalized NMR data were UV‐scaled prior to multivariate analysis. Multivariate data analysis methods, principal component analysis (PCA), and orthogonal partial least squares discriminant analysis (OPLS‐DA) were used to reduce the dimensionality and to enable the visualization of the separation of diet types (SIMCA 14.0, Umetrics). An unsupervised PCA was performed to obtain a trend of separation of samples according to groups (e.g., sex, diet type, and pregnancy status) and identified possible outliers. To maximize the sample group separation and identify discriminating metabolites, OPLS‐DA analysis was carried out. This supervised approach removes the variation that is orthogonal to predefined variables from the models that were calculated for each two‐group comparison, making them easier to interpret and thus more informative. All OPLS‐DA models were described by the number of principal components, the amount of variation in X explained by the model (R^2^X), the amount of variation in Y explained by the model (R^2^Y), the amount of variation in Y predicted by the model (Q^2^). To assess the predictive ability of the models, a sevenfold cross‐validation was used. Further validation of the models was carried out by using cross‐validation ANOVA (CV‐ANOVA) and inspection of corresponding permutations plots. Important metabolites differentiating selected groups were selected based on model covariance loadings (|w*| ≥ 0.15) from respective OPLS‐DA model and results of univariate analysis using *t* test with Benjamini–Hochberg correction (*p* ≤ 0.05) were used to determine significantly altered metabolites. Simplified representation of metabolic pathways was based on KEGG Pathway Database (http://www.genome.jp/kegg/pathway.html).

## 3 RESULTS

### 3.1 Dietary profiles

Our DNA‐metabarcoding approach resulted in the detection of 17 molecular operational taxonomic units (MOTUs) in moose winter diet (Appendix, Table A1). The number of MOTUs detected per individual sample ranged from 1 to 12. One of 265 fecal samples did not pass the filtering criteria in the metabarcoding process and was excluded from subsequent analyses.

**Figure 2:**
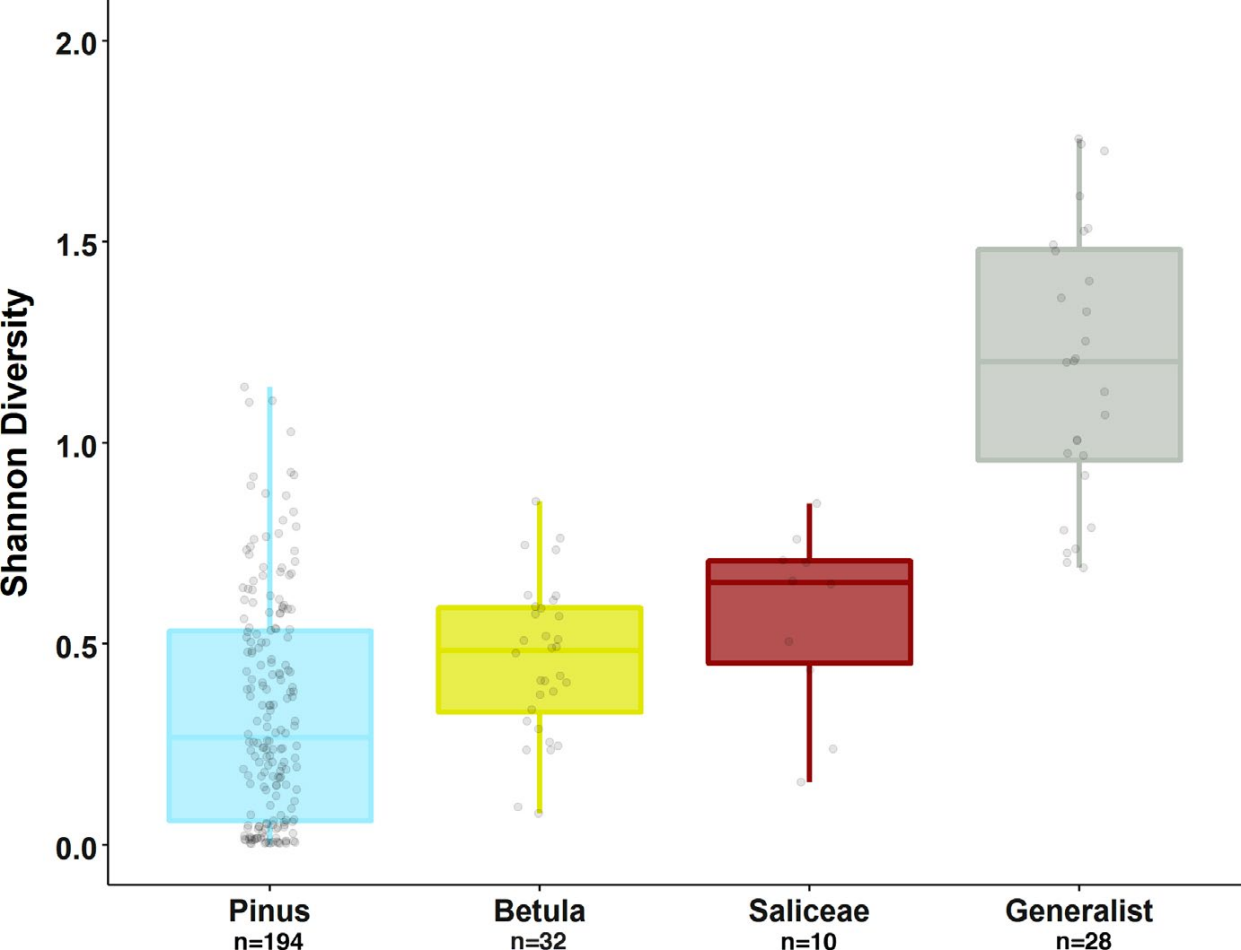
Diet diversity of 264 moose winter diets categorized by diet types based on 17 MOTUs. Diet types are categorized based on individuals consuming more (“specialists”: *Pinus*—turquoise, *Betula*—yellow, and Saliceae—burgundy) or less (“generalist”—gray) than 60% of a single diet item

The 60%‐threshold to differentiate “specialist” from “generalist” diets, resulted in four categories of diet types (Figure 1). The majority of moose exhibited a “specialist” diet, with 194 individuals having a pine‐rich (*Pinus*) diet, while 32 individuals had diets dominated by birch (*Betula*) and 10 that were dominated by Saliceae, that is,. willow and aspen. A “generalist” diet type was found in 28 individuals (11%). Pine‐rich diets were least diverse, while “generalist” diets were most diverse (Figure 2).

**Figure A2:**
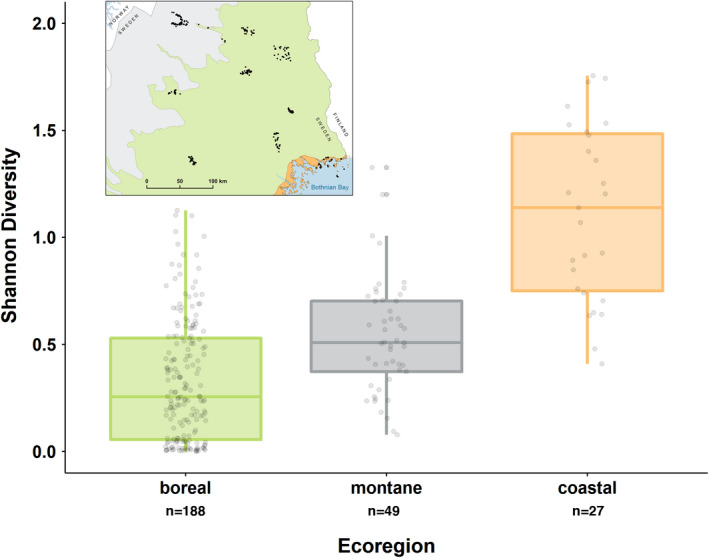
Diversity of 264 moose winter diets based on the ecoregion (corresponding to boxplot shades) that individuals were captured in

Diet types reflect ecozonation and further, the land use intensity of moose winter range (Figure 1; Appendix, Figures A1 and A2), thereby representing the availability of tree species during the dormant season. Half (52%) of the generalist moose diets occurred in the coastal ecoregion, whereas diets of animals captured in the montane region were dominated by birch (61%). While 96% of diets from the boreal region consumed pine‐rich diets, this diet type accounts for 41% in the coastal and only approximately 6% in the montane region, respectively. The proportions of generalist and willow/aspen‐rich diet in the montane are at an equal 16%.

**Figure A3:**
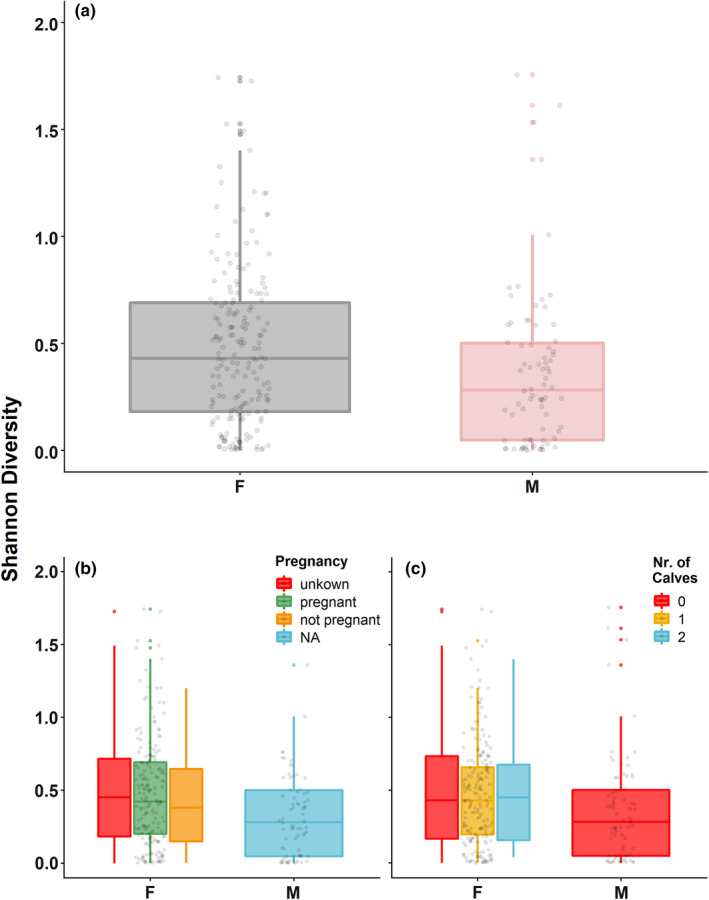
Diet diversity of 264 moose winter diets based on 17 MOTUs and distinguished by sex (A; 186 females, 78 males). Neither pregnancy status (B) nor the number of calves (C) is driving the significant (*p* = .004) difference between sexes

A nonparametric two‐samples Wilcoxon rank test determined a significant difference (*p* = 0.004) of diet diversity between sexes (Appendix, Figure A3). Females exhibited a higher (and more variable) diet diversity and consumed proportionally less pine than males. This difference was neither explained by pregnancy state nor by the number of offspring accompanying the mothers.

### 3.2 Metabolomic profiles

**Figure A4:**
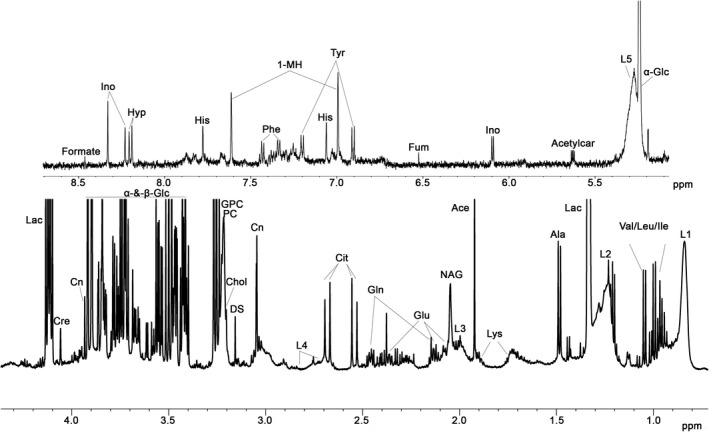
Representative 600‐MHz 1H CPMG NMR spectrum of moose serum. The regions of δ 5.1–8.7 (above) were magnified compared with the corresponding regions δ 0.6–4.5 (below) for purposes of clarity. Ino: inosine, Hyp: hypoxanthine, His: histidine, 1‐ MH: 1‐methylhistidine, Phe: phenylalanine, Tyr: tyrosine, Fum: fumarate, Acetylcar: acetylcarnitine, L5: lipid –HC = CH, α‐Glc: α‐glucose, Lac: lactate, Cre: creatine, Cn: creatinine, α‐&‐ß‐Glc: α‐&‐ß‐glucose, PC: phosphocholine, GPC: glycerophosphocholine, Chol: choline, DS: dimethyl sulfone, L4: lipid C = CCH2C=C, Cit: citrate, Gln: glutamine, Glu: glutathione, NAG: N‐acetylglycoprotein, L3: lipid CH2C = C, Ace: acetate, Lys: lysine, Ala: alanine, L2: lipid (CH2)n, Val: valine, Leu: leucine, Ile: isoleucine, L1: lipid –CH3

Nontargeted metabolomics analysis with application of ^1^H NMR spectroscopy was performed for 260 serum samples. Four serum samples did not pass the quality criteria required for ^1^H NMR analysis and were therefore removed from subsequent analysis. A representative 600 MHz ^1^H CPMG NMR spectrum of a serum sample is presented in Appendix, Figure A4. Due to the well resolved individual NMR resonances, a wide variety of metabolites can be differentiated and putatively identified. In total, 55 metabolites were putatively identified and quantified (complete list of identified metabolites is shown in Appendix, Table S2). The most predominant metabolites were as follows: BCAA (branched‐chain amino acids), alanine, lactate, lysine, acetate, glutamate, glutamine, pyruvate, citrate, creatinine, glycerophosphocholine, phosphocholine, glycine, urea, tyrosine, phenylalanine, 1‐methylhistidine, formate, and numerous lipid species.

**Figure A5:**
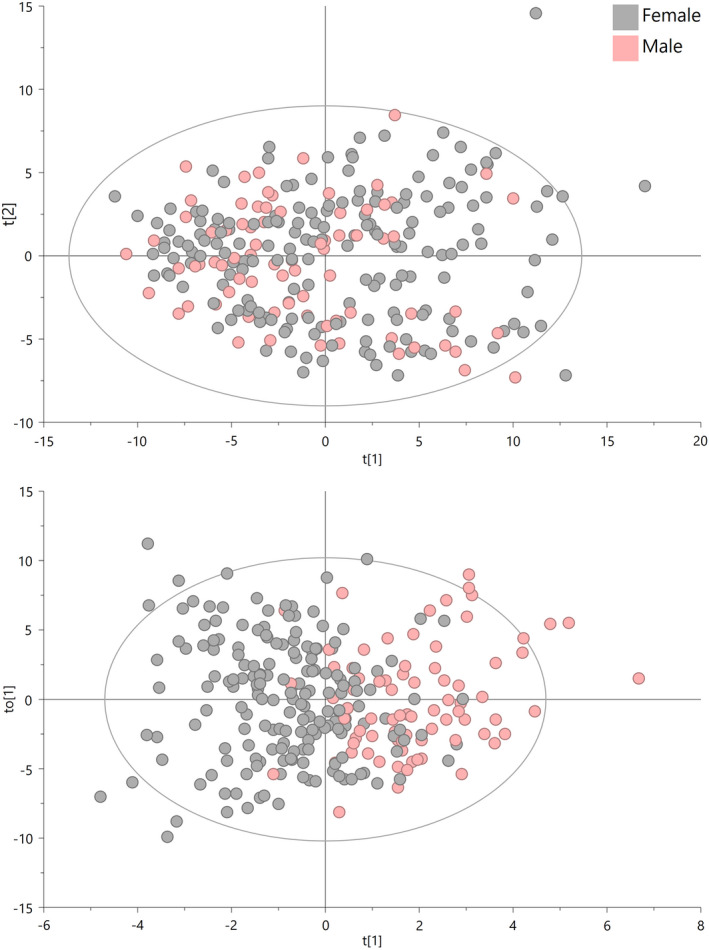
Serum metabolomics multivariate analysis of two‐way comparison of sexes (gray circles = females; pink circles = males). PCA (above) and OPLS‐DA (below) score plots for comparison of sex differences. Hostelling's ellipse delineates the confidence at 95% of the distance of the scores to the mean values

Metabolomic profiles were different between sexes as illustrated in an OPLS‐DA score plot (Appendix, Figure A5). Goodness of fit values and predictive ability values were obtained (R^2^X = 0.529, R^2^Y = 0.443, Q^2^ = 0.29) indicating that the model had a reasonably good fit and predictive power. A CV‐ANOVA showed highly significant variation related to the separation of groups, *p*‐value <0.01. Based on the |w*|‐vales from the OPLS‐DA model we selected ten metabolites differing between sexes. However, only creatinine and albumin lysyl were statistically significant (*t* test *p*‐value ≤ 0.05) and males exhibited decreased concentrations of both (Appendix, Table A3). Metabolomic patterns did not differ between pregnant and nonpregnant females or between females with and without accompanying offspring.

### 3.3 Linking dietary and metabolomics profiles

**Figure 3:**
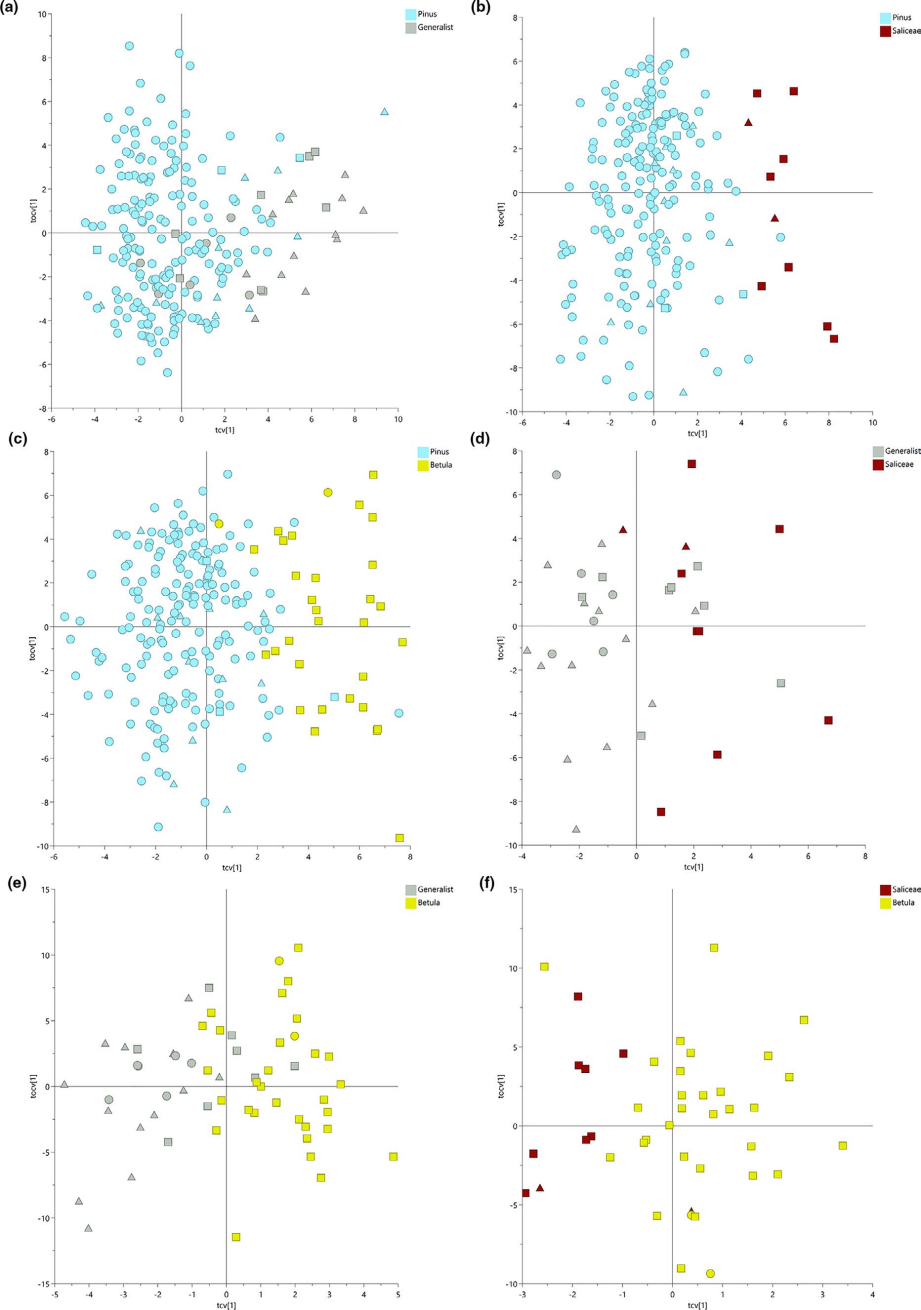
Serum metabolomics multivariate analysis of two‐way comparison of diet types. Cross‐validated score plots of OPLS‐DA models for each comparison. (a) *Pinus* versus Generalist, (b) *Pinus* versus Saliceae, (c) *Pinus* versus *Betula*, (d) Generalist versus Saliceae, (e) Generalist versus *Betula*, (f) Saliceae versus *Betula*. Ecoregions were marked by shape symbols: squares—montane, circles—boreal, triangles—coastal

**Figure A6:**
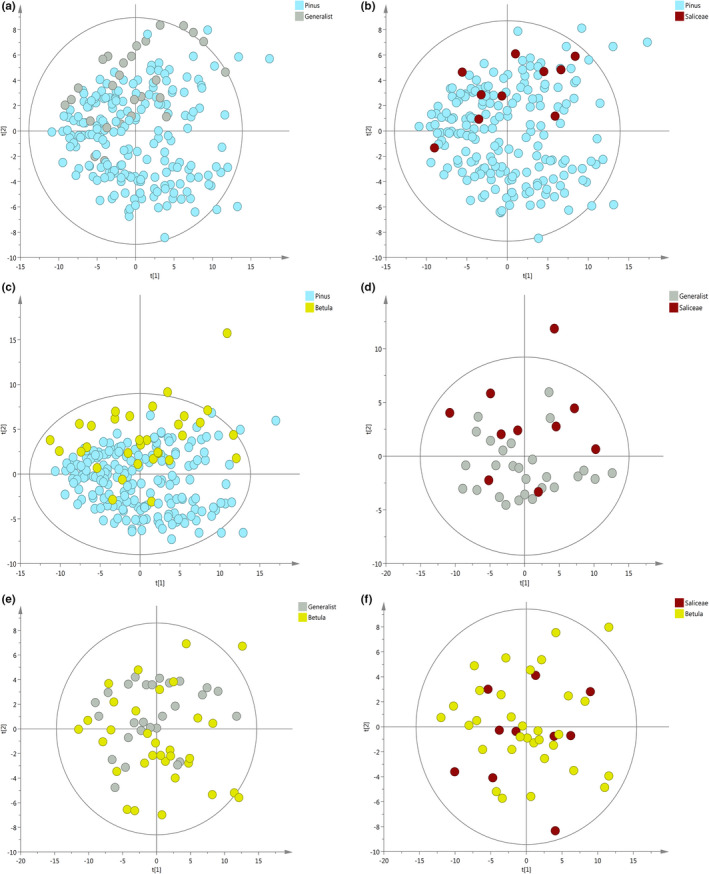
Serum metabolomics multivariate analysis of two‐way comparison of diet types. PCA score plots for each comparison, the lined ellipse represents 95% confidence intervals. A. *Pinus* vs. Generalist, B. *Pinus* vs. Saliceae, C. *Pinus* vs. *Betula*, D. Generalist vs. Saliceae, E. Generalist vs. *Betula*, F. Saliceae vs. *Betula*

To investigate the metabolic variations correlated with four diet types, we conducted a two‐way comparison of each diet type, obtaining six comparisons in total. First, PCA plots were investigated, presenting an unclear separation of diet‐groups (Appendix, Figure A6). Second, OPLS‐DA models were established for each of comparison to clearly discriminate and identify metabolites based on diet types (Figure 3).

**Figure A7:**
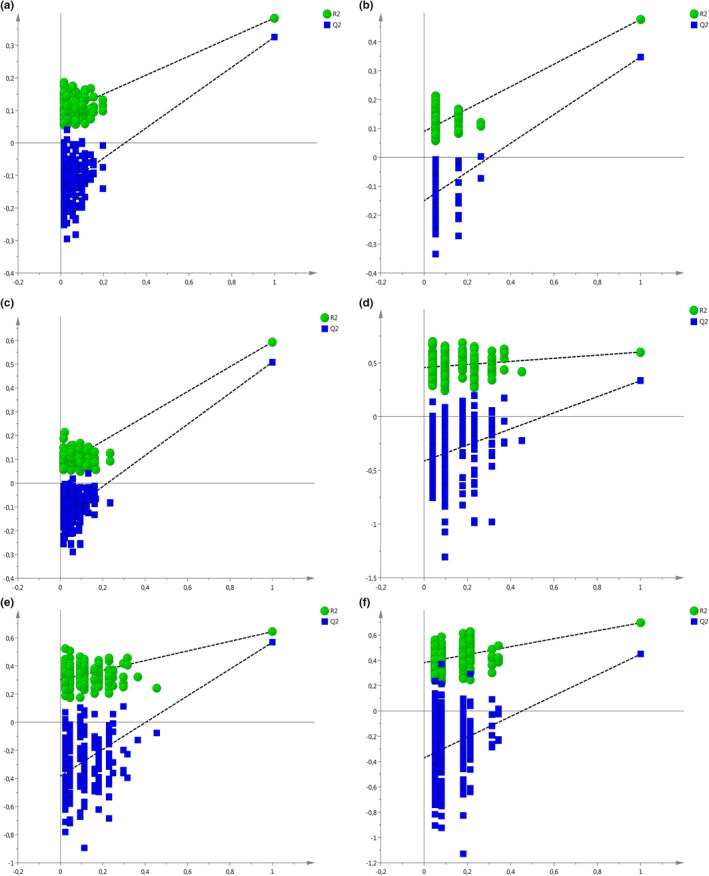
Plots obtained after performing a random permutation test with 200 permutations on OPLS‐DA models of 1H NMR data. R2 is the explained variance, and Q2 is the predictive ability of the model. Low value of Q2‐intercept depicts the high predictability of the model. A. *Pinus* vs. Generalist, B. *Pinus* vs. Saliceae, C. *Pinus* vs. *Betula*, D. Generalist vs. Saliceae, E. Generalist vs. *Betula*, F. Saliceae vs. *Betula*

Detailed characterization of two‐class OPLS‐DA models obtained based on ^1^H NMR metabolomic data distinguished by moose winter diet types is summarized in Appendix, Table A4. The permutation tests confirmed the robustness of all OPLS‐DA models (Appendix, Figure A7).

Linking metabolic profiles with diet types resulted in the identification of 29 metabolites that significantly drove the separation of ^1^H NMR metabolomic profiles among four diet types (as seen in Figure 3). A pathway network map of significantly altered metabolites, including box plots of metabolites for four different diet types, summarizes the key significant metabolites that are major intermediates of pathways involving amino acid, lipid, and gut microbiota metabolism. The following pattern of decreased levels of lipids (specifically the fatty acids ‐CH_3_ (HDL), CH_3_(CH_2_)_n_, CH2‐C = C, ‐CH = CH‐), glycerophosphocholine, and phosphocholine in animals with a birch‐ (*Betula*) and willow/aspen‐rich diet (Saliceae) (that dominate the montane and coastal ecoregion, respectively) compared to pine‐rich (*Pinus*) diets was observed.

The reverse trend was observed for amino acids, such as glutathione, threonine, proline, phenylalanine, tyrosine, glycine, histidine, and ornithine. They all were decreased in pine‐rich diets (*Pinus*) with respect to all other types of diet (*Betula*, Saliceae, and Generalist). Moreover, differences in gut microbiota were directly reflected by a significant increase of dimethyl sulfone in moose with pine‐rich diets (*Pinus*) compared to all other types of diet (*Betula*, Saliceae, and Generalist).

**Figure 4:**
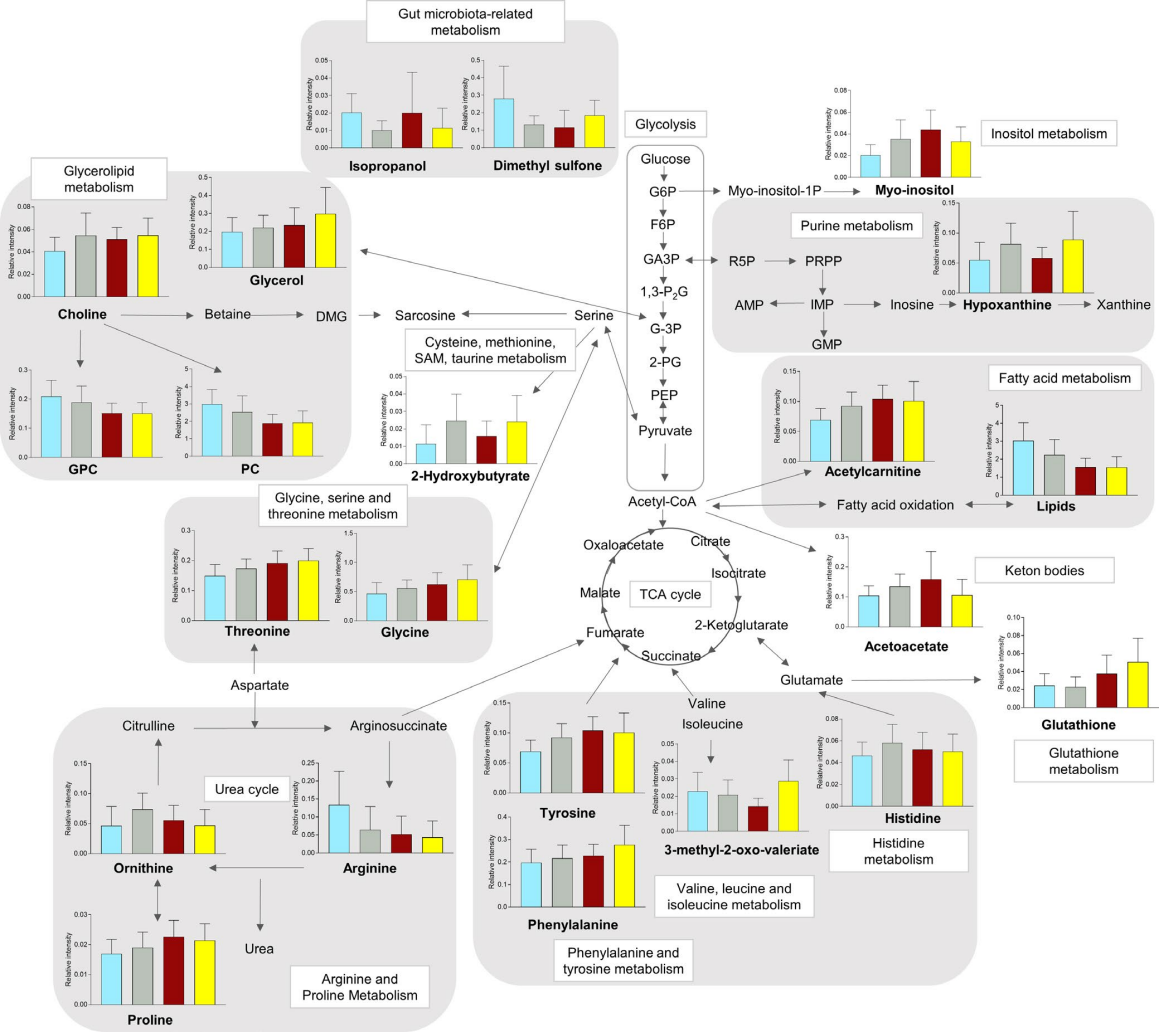
Simplified representation of metabolic pathways showing significantly altered metabolites in four categories based on moose diet types: *Pinus* (turquoise bars), Generalist (gray bars), Saliceae (burgundy bars), *Betula* (yellow bars)

Additionally, we observed significant differences in the levels of two metabolites related to energy metabolism. Acetoacetate was significantly elevated in individuals that consumed a generalist diet compared to a pine‐rich diet, whereas animals that exhibit a generalist and birch‐rich diet compared with pine‐rich diet exhibited elevated levels of 2‐Hydroxybutyrate (Table 1; Figure 4).

**Table 1:** Metabolites driving separation of ^1^H NMR metabolomic profiles among moose diet types determined by multivariate and univariate analyses, corresponding to metabolites illustrated in Figure 4

Metabolite	Pathway	Superpathway	Generalist versus Pinus		Saliseae versus Pinus		Betula versus Pinus		Saliceae versus Generalist		Betula versus Generalist		Betula versus Saliceae	
				*p*‐value		*p*‐value		*p*‐value		*p*‐value		*p*‐value		*p*‐value
Lipids ‐CH_3_ (HDL)	Fatty Acid Metabolism	Lipid	↓	0.052	↓	***0.001***	↓	***<0.001***	↓	0.182	↓	***0.040***	↓	0.968
Lipids CH_3_(CH_2_)*_n_*	Fatty Acid Metabolism	Lipid	↓	***0.001***	↓	***<0.001***	↓	***<0.001***	↓	0.182	↓	***0.019***	↓	0.979
Lipids CH_2_‐C = C	Fatty Acid Metabolism	Lipid	↓	0.331	↓	**0.005**	↓	***<0.001***	↓	0.182	↓	0.070	↑	0.968
Lipids = C‐CH_2_‐C=	Fatty Acid Metabolism	Lipid	↑	0.285	↑	***0.004***	↑	**<0.001**	↑	0.182	↑	0.214	↓	0.727
Lipids –CH = CH‐	Fatty Acid Metabolism	Lipid	↓	**0.001**	↓	***<0.001***	↓	***<0.001***	↓	0.215	↓	***0.016***	↓	0.727
Acetylcarnitine	Fatty Acid Metabolism (Acyl Carnitine)	Lipid	↑	***0.001***	↑	0.608	↑	***<0.001***	↓	2.88	↑	0.681	↑	0.513
Choline	Phospholipid Metabolism	Lipid	↑	***0.006***	↑	**0.048**	↑	***<0.001***	↓	0.764	↑	0.997	↑	0.805
Phosphocholine	Phospholipid Metabolism	Lipid	↓	**0.044**	↓	***0.001***	↓	***<0.001***	↓	0.182	↓	0.059	↑	0.968
Glycerol	Glycerolipid metabolism	Lipid	↑	0.216	↑	0.461	↑	***0.002***	↑	0.826	↑	0.071	↑	0.688
Glycerophosphocholine	Glycerolipid metabolism	Lipid	↓	0.150	↓	***0.004***	↓	***<0.001***	↓	0.235	↓	0.052	↓	0.979
Myo‐inositol	Inositol metabolism	Lipid	↑	***0.001***	↑	***0.015***	↑	***<0.001***	↑	0.520	↓	0.727	↓	0.683
Acetoacetate	Ketone bodies	Lipid	↑	***0.004***	↑	0.228	↑	0.866	↑	0.764	↓	0.117	↓	0.683
Tyrosine	Phenylalanine & tyrosine metabolism	Amino acid	↑	***<0.001***	↑	***0.005***	↑	***<0.001***	↑	0.520	↑	0.519	↓	0.940
Phenylalanine	Phenylalanine & tyrosine metabolism	Amino acid	↑	0.182	↑	0.217	↑	***<0.001***	↑	0.793	↑	***0.045***	↑	0.624
2‐Hydroxybutyrate	Cysteine, methionine, SAM, taurine metabolism	Amino acid	↑	***0.001***	↑	0.290	↑	***<0.001***	↓	0.288	↓	0.988	↑	0.624
Histidine	Histidine metabolism	Amino acid	↑	***0.007***	↑	0.461	↑	0.320	↓	0.619	↓	0.308	↓	0.968
Proline	Urea cycle; Arginine and Proline Metabolism	Amino acid	↑	0.109	↑	***0.042***	↑	**0.001**	↑	0.372	↑	0.283	↓	0.855
Arginine	Urea cycle; Arginine and Proline Metabolism	Amino acid	↓	**<0.001**	↓	**0.004**	↓	***<0.001***	↓	0.770	↓	0.382	↓	0.912
Ornithine	Urea cycle; Arginine and Proline Metabolism	Amino acid	↑	***<0.001***	↑	0.461	↑	0.947	↓	0.300	**↓**	***0.015***	↓	0.788
Threonine	Glycine, Serine, and Threonine Metabolism	Amino acid	↑	**0.003**	↑	***0.037***	↑	**<0.001**	↑	0.520	↑	0.059	↑	0.876
Glycine	Glycine, serine, and threonine metabolism	Amino acid	↑	**0.019**	↑	0.110	↑	**<0.001**	↑	0.619	↑	0.052	↑	0.727
Glutathione	Glutathione Metabolism	Amino acid	↓	0.633	↑	0.185	↑	**<0.001**	↑	0.288	↑	***< 0.001***	↑	0.683
3‐Methyl‐oxo‐valeriate	Valine, leucine, and isoleucine metabolism	Amino acid	↓	0.329	↓	**0.002**	↑	**0.037**	↓	0.764	↑	0.727	↑	***< 0.001***
Hypoxanthine	Purine metabolism, (hypo)xanthine/ inosine containing	Nucleotide	↑	***0.003***	↑	0.730	↑	***0.001***	↓	0.182	↑	0.727	↑	0.218
Isopropanol		Gut microbiota	↓	***<0.001***	↓	0.993	↓	**0.001**	↑	0.520	↑	0.727	↓	0.727
Dimethyl sulfone		Gut microbiota	↓	***<0.001***	↓	**0.004**	↓	**<0.001**	↓	0.826	↑	0.059	↑	0.683
Unknown *1.36 ppm*	‐	‐	↓	***<0.001***	↓	**0.027**	↓	0.78	↓	0.826	↑	0.117	↑	0.683
Unknown *2.06 ppm*	‐	‐	↑	***<0.001***	↑	**0.022**	↑	0.016	↑	0.628	↓	0.519	↓	0.683
Unknown *6.81 ppm*	‐	‐	↑	***0.001***	↑	0.777	↑	0.131	↓	0.179	↑	0.664	↑	0.683

Note

*p*‐values are a result of a *t* test with Benjamini–Hochberg correction (*p* ≤ 0.05).

Values marked on bold indicate significance and metabolites marked in cursive additionally have a |w*|‐value ≥ 0.15 based on the two‐class OPLS‐DA model corresponding to each *t* test.

**Figure A8:**
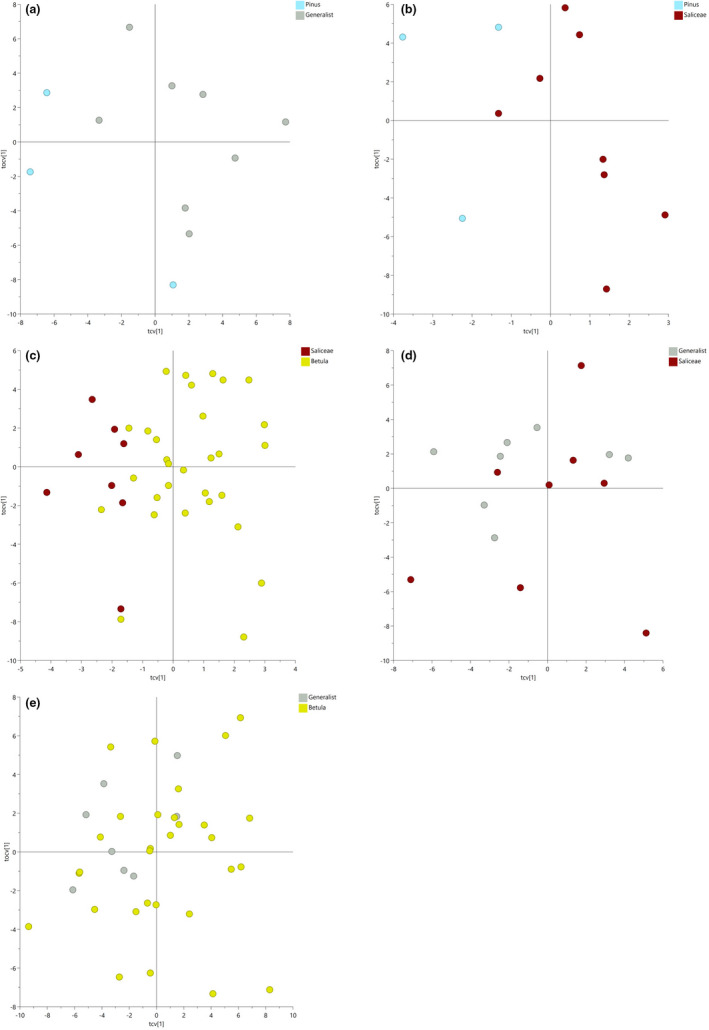
Serum metabolomics multivariate analysis of two‐way comparison of diet types only in montane ecoregion. Cross‐validated score plots of OPLS‐DA models for each comparison. A. *Pinus* vs. Generalist; (1 + 1 components), R2X(cum) = 0.583, R2Y(cum) = 0.646, Q2(cum) = 0.389, B. *Pinus* vs. Saliceae; (1 + 1 components), R2X(cum) = 0.549, R2Y(cum) = 0.941, Q2(cum) = 0.527, C. Generalist vs. Saliceae; (1 + 1 components), R2X(cum) = 0.530, R2Y(cum) = 0.345, Q2(cum) = 0.0454, D. Generalist vs. *Betula*; (1 + 1 components), R2X(cum) = 0.472, R2Y(cum) = 0.208, Q2(cum) = 0.0833, F. Saliceae vs. *Betula*; (1 + 1 components), R2X(cum) = 0.234, R2Y(cum) = 0.698, Q2(cum) = 0.433

We attempted additional validation of our results by analyzing diet type classification within the montane region since all four diet types were present in this ecoregion via OPLS‐DA score plots of two‐way comparison (Appendix, Figure A8). A *Betula* versus *Pinus* model could not be obtained for a comparison. The resulting classification for the montane ecoregion did not perform as well as for models including all regions with higher number of samples. However, grouping of specific diet types could still be observed.

## 4 DISCUSSION

Our results provide important baseline physiological information on a large herbivore species that is widely distributed and is adapted to diverse habitat conditions across a large latitudinal gradient in the northern hemisphere. We demonstrate the adaptive flexibility of moose in terms of dietary use and we identified important biomarkers that provide vital hints on linkages between its diet and the potential to adapt physiologically to extreme and wide variety of climatic and land use conditions.

Using a ^1^H NMR approach, we were able to show that serum metabolomic profiles of moose varied among the four different diet types. These diet types clearly mirrored the ecozonation and were further characterized by the land use (see Figure 1), adding to evidence of a high degree of flexibility that moose exhibit (Hoy et al., 2019; Vivas and Saether, 1987; Parikh et al., 2017). We confirm that moose unavoidably experience a nutritional deficit due to limiting forage availability during winter. However, the degree of this nutritional deficit seems to be highly variable between areas, as we show by identifying biomarkers of starvation in moose blood.

### 4.1 Dietary profiles

We broadly identified four diet types in moose winter diet as “specialist” pine‐rich, birch‐rich, Saliceae‐rich and a more diverse “generalist” diet. These diet types not only reflect the ecozonation of moose winter range but also correspond to the dominant type of land uses in the area, which influences the vegetation and the forage availability. The major land use in Northern areas of Sweden is forestry and other types of extractive industry such as mining (Fohringer et al. in review). Even though our samples reflect the climatic coast to mountain gradient, they also are affected by intensive forestry practices manipulating the supply of forage species to moose, especially the quantity of Scots pine which is the major timber species.

The quantity of pine varies across our sampling gradient with minimum abundance in the montane region (hence, a birch‐rich diet in moose in this area) and the coastal protected area region (more diverse or generalist diet) and highest in the boreal zone (evident from the pine‐rich diet in moose from this area). There are other mechanisms such as disturbance and habitat loss potentially triggered by mining, tourism, and interference that may indirectly affect moose habitat choice and their metabolic expenditure as well (Fohringer et al., in review).

We found differences in diet diversity across sexes. However, among females, we found no effect of pregnancy status or the presence of offspring on diet diversity (Appendix, Figure A3). The differences between the sexes can be attributed to a higher mean proportion of *Pinus* consumed by males, as range use in our study area is not significantly different between the sexes (see Allen, Dorey, et al., 2016). This difference follows the Jarman‐Bell principle (Geist, 1974) enabling the generally larger sized males to rely less on quality but rather on abundant diet items during winter. Moose males generally enter winter with lower energy reserves as they spend less time foraging during the rutting period (late September to mid‐October) compared to females. Hence, males should typically maximize their instantaneous rate of food intake to sustain them through the winter (Du Toit, 2006).

### 4.2 Metabolomic profiles

Similar to the separation in diet types, and perhaps also driven by this, we found differences in energy metabolism of individuals based on the biomarkers observed across ecoregions. Pine‐rich diets had more lipids in moose serum than the other diets whereas amino acids and ketone bodies were higher in the birch and willow diets than in the pine diets. The generalist diets were elevated in ketone bodies relative to the other diets. The elevated concentrations of lipids indicate short periods of starvation, whereas increased concentrations of amino acids and ketone bodies indicate prolonged starvation. Again, this points to the fact that climatic and land use patterns may affect animal physiology through the manipulation of forage species and thereby affecting diet choice (Allen, Månsson, et al., 2016). Metabolic shifts are unlikely attributed to genetic differences among animals, since individuals included in our study area were found to be genetically similar (Blåhed et al., 2018; Niedzialkowska et al., 2016; Wennerstrom et al., 2016). When animals experience food deprivation, they respond by mobilizing internal energy stores, triggering a starvation response. In early starvation of vertebrates, stores of glycogen and triglyceride provide most of the metabolic needs of tissues. Fatty acids are being released from liver and adipose for use in other tissues. After prolonged starvation, glycogen reserves are depleted and ketone bodies are produced from fatty acids from adipose and liver, as well as some amino acids derived from muscle proteolysis (Moyes & Schulte, 2008).

Another important set of biomarkers, we found were serum creatinine and albumin lysyl which are known to be associated with muscle mass and nutritional restriction, respectively, especially from studies in humans (Lee et al., 2015; Schutte et al., 1981). We found the concentrations of these to be different between the sexes (lower in males), potentially indicating differences in the ecology of the sexes, when males lose more muscle mass during rut than the females and simultaneously compromising on nutritional gains by feeding less (Miquelle, 1990; Mysterud et al., 2005). Therefore, one would expect that these differences would not persist across ecoregions or across land use pressures unless these disrupt the reproductive ecology of the species locally. Rather, changes in sex ratio as well as density dependence may bring about differences across regions or in time, linked to reproductive collapse through hunting (e.g., Milner‐Gulland et al., 2003). Nevertheless, we only present spatial trends and not temporal, in this study.

### 4.3 Linkages

Animals consuming predominantly *Pinus* exhibit significantly higher dimethyl sulfone concentrations compared with all other diet types. This compound is derived from dietary sources, intestinal bacterial metabolism, and the body's endogenous methanethiol metabolism (He & Slupsky, 2014).

The presence of dimethyl sulfone in human biofluids has been shown to be highly influenced by diet and a high content thereof is suggested to explain enhanced intestinal fermentation activity due to fiber consumption (He & Slupsky, 2014). In ruminants, several studies have attributed increased levels of dimethyl sulfone to increased levels of dietary crude protein (Coppa et al., 2011; O’Callaghan et al., 2018). This hints toward the fact that even though moose in pine‐dominated areas are limited in diet diversity, they may still be able to cope well due to high dimethyl sulfone promoting fermentation. This redirects back to the observations that moose being large and bulk feeders may be able to survive on abundant, relatively low quality, forage (pine being the most abundant browse species in Fennoscandia) instead of depending on high‐quality forage, especially in lean winter months (Felton et al., 2020; Månsson et al., 2007; Pfeffer et al., 2021). Conversely, animals that have not consumed this bulk diet item extensively—most likely due to restricted availability—may not be able to provide their fermentative bacteria with sufficient cellulose, thus, reducing microbial activity in the rumen which is reflected in significantly lower concentrations of dimethyl sulfone in their serum. Animals in such regions are likely to perform worse in the winters which would be reflected in the variable concentrations of other metabolites such as lipids that may indicate starvation.

Higher diet diversity is suggested to be beneficial for moose to reduce the risk of overconsumption of secondary plant metabolites (Felton et al., 2020; Parikh et al., 2017). It is difficult to conclude, at what level does the diet diversity becomes crucial, and what is the tolerance boundary for moose for that. We may conclude though, that pine may serve as a bulk item that is “good enough,” providing endosymbiotic bacteria with sufficient amounts of cellulose to keep beneficial dimethyl sulfone at a comparatively higher level.

Increased concentrations of ketone bodies, such as acetoacetate, present in individuals that consumed a generalist diet compared with a pine‐rich diet, indicate late starvation or prolonged physical exertion as part of gluconeogenesis (Hogan & Philips, 2016; Leng, 1970). We found increased 2‐hydroxybutyrate for animals that exhibit a generalist and birch‐rich diet compared with those that had a pine‐rich diet. This metabolite generally appears at high concentrations in situations related to impeded energy metabolism and was suggested to be an early marker indicating impaired glucose regulation that appears to arise due to increased lipid oxidation and oxidative stress in humans (Gall et al., 2010). If this also applies in moose, the identification of such biomarkers of stress becomes an important finding. This is especially applicable in the context of environmental stress related to climate and land use changes, since moose in montane regions facing extreme snow conditions along with extensive starvation bouts and those in coastal areas facing human land use pressure reducing access to certain key forage species, may experience oxidative stress beyond baseline conditions. Conversely, extensive forest management in the boreal region creates abundant supply of pine, a staple food source for moose, thereby decreasing stress and starvation.

We have identified and linked several important biomarkers in this study (Figure 4) and there are yet numerous more which we have found but not discussed (Appendix, Table A2).These warrant careful considerations and analyses, and here, we provide a blueprint on how one can go about exploring these further and linking to various other aspects of animal physiology and ecology. A caveat of our study is the potential confounding effect of ecoregion, which encompasses both climate and vegetation, hence, representing forage availability and thermal environment for an animal. Diet composition on the other hand represents habitat selection of an animal which is in this case, again influenced by the ecoregion. After conducting multivariate analysis within the only ecoregion where all four diet types were present, clustering of diet types remained but was less striking compared to the same analysis across all ecoregions. Despite, ruling out potential population effects, we can therefore not rule out that other environmental factors encompassed by ecoregions (beyond diet) also influence metabolic profiles. Moreover, we may have missed to identify biomarkers of animal health, behavior and fitness, and mechanisms driving these due to the absence of relevant ancillary information to test for these parameters. Calls have been made by recent reviews for the high potential of the integration of multi‐omics approaches to facilitate a more holistic understanding of how organisms respond to different stressors (Gooseens et al., 2020; Schwartz, 2020).

## 5 CONCLUSION

We show how NMR‐based metabolomics can be used to identify molecules that give novel insights on species ecophysiology. Our study exemplifies that the integration of omics approaches into routinely used monitoring protocols is not only feasible but also combinable, even in nonexperimental nonclinical set‐ups such as the one examined. Most other studies on free‐ranging vertebrates have evaluated feasibility and have not gone as far as to make functional linkages between physiology and diet at this resolution. Integrative approaches bridging biomolecular methods with animal demographics and environmental variables to elucidate the effects of diet on organismal metabolism and vice versa are essential to understand diet choices and shifts and corresponding physiological responses across the species range.

We see a great potential for the application of our combined omics approach for longitudinal studies to detect seasonal and annual shifts in metabolism and diet in association with movement characteristics, fitness proxies, and environmental data. In addition to nonlethal serum sampling, alternative bioliquids, such as rumen fluid or liquefied feces can be analyzed in future environmental omics studies to attain even deeper insight into metabolic consequences of environmental change. The biomarkers (metabolites) identified by our approach are intended to encourage subsequent monitoring by means of additional diagnostics assays (e.g., lipidomics, proteomics, metallomics, stable isotope analysis, or hormone assays) that can then be associated with animal diets, their microbiome, physiology, and energy metabolism in further detail.

## CONFLICT OF INTERESTS

None declared.

## AUTHOR CONTRIBUTIONS

**Christian Fohringer:** Conceptualization (lead); Data curation (equal); Funding acquisition (equal); Methodology (equal); Validation (lead); Visualization (lead); Writing‐original draft (lead); Writing‐review & editing (lead). **Ilona Dudka:** Conceptualization (equal); Data curation (equal); Formal analysis (equal); Methodology (equal); Software (equal); Validation (equal); Visualization (equal); Writing‐original draft (equal); Writing‐review & editing (equal). **Robert Spitzer:** Data curation (supporting); Methodology (equal); Validation (equal); Writing‐review & editing (equal). **Fredrik Stenbacka:** Methodology (equal); Validation (supporting); Writing‐review & editing (equal). **Olena Rzhepishevska:** Conceptualization (equal); Writing‐review & editing (equal). **Joris P.G.M. Cromsigt:** Funding acquisition (equal); Writing‐review & editing (equal). **Gerhard Gröbner:** Methodology (supporting); Writing‐review & editing (equal). **Göran Ericsson:** Supervision (supporting); Writing‐review & editing (equal). **Navinder Singh:** Conceptualization (equal); Supervision (equal); Writing‐original draft (equal); Writing‐review & editing (equal).

## Data Availability

Data are available from the Dryad Digital Repository: https://doi.org/10.5061/dryad.9s4mw6mfr

## Appendix 1

**Table A1:** Plant species identification and classification of molecular operational taxonomic units (MOTUs) of moose fecal samples based on DNA‐metabarcoding data determined at a threshold of 2.5%

MOTUs [*N* = 17]	Taxonomic rank	Presumed taxon^a^
*Alnus*	genus	***A. incana***
*Betula*	genus	***B. pubescens*, *B. pendula***, *B. nana*
*Juniperus*	genus	***J. communis***
*Picea*	genus	***P. abies***
*Pinus*	genus	***P. sylvestris*,** *P. contorta*
Saliceae	tribe	***P. tremula*** and multiple species of the genus *Salix*
*Populus* ^b^	genus	
*Pinus contorta* ^c^	species	
*Empetrum*	genus	***E. nigrum***
Fagales	order	Potentially many genera, incl. *Alnus* and *Betula*
*Arctostaphylos uva‐ursi*	species	
Rosales	order	***Sorbus aucuparia***, *Rubus*
*Filipendula ulmaria* ^d^	species	
*Rumex*	genus	*R. acetosa*, *R. longifolius*
*Prunus* ^d^	genus	***P. padus***
Solanoideae	subfamily	*Solanum tuberosum*
Triticeae	tribe	

^a^ Referenced via ‘https://artfakta.se/artbestamning’ and ‘http://linnaeus.nrm.se/flora/’ (last accessed: 06/10/2020). Species indicated in bold are prime candidate species based on distribution and likelihood of availability during winter.

^b^ Collapsed into Saliceae (distinction between *Salix* and *Populus* not possible; both genera constitute Saliceae).

^c^ Collapsed into *Pinus* (because *P. contorta* could be included in *Pinus*).

^d^ Collapsed into Rosales (because they could be included in Rosales).

**Table A2:** Metabolites driving the separation of ^1^H NMR metabolomic profiles among moose winter diet types determined by multivariate and univariate analyses

Metabolite	Chemical shift (ppm)	Pathway	Superpathway
Formate	8.46		Gut microbiota
Inosine/Adenosine	**8.32**, 8.22, 6.09	Purine metabolism, (hypo)xanthine/ inosine containing	Nucleotide
Hypoxanthine	8.19	Purine metabolism, (hypo)xanthine/ inosine containing	Nucleotide
Histidine	**7.77**, 7.06	Histidine metabolism	Amino acid
1‐Methylhistidine	7.60, **6.99**, 4.04, 3.68	Histidine metabolism	Amino acid
Phenylalanine	7.38	Phenylalanine & tyrosine metabolism	Amino acid
Tyrosine	7.20, **6.90**	Phenylalanine & tyrosine metabolism	Amino acid
Fumarate	6.52	Krebs cycle	Energy
Acetylcarnitine	5.63	Fatty Acid Metabolism (Acyl Carnitine)	Lipid
Lipids –CH = CH‐	5.31	Fatty Acid Metabolism	Lipid
α‐Glucose	**5.24**, 3.81, 3.47, 3.26	Glycolysis, Gluconeogenesis, and Pyruvate Metabolism	Carbohydrate
Mannose	**5.19**, 3.95	Fructose, Mannose and Galactose Metabolism	Carbohydrate
Glycerophosphocholine	**4.32**, 3.95, 3.66, 3.21	Glycerolipid metabolism	Lipid
Threonine	4.24	Glycine, Serine, and Threonine Metabolism	Amino acid
Pyroglutamate (5‐oxoproline)	**4.19**, 2.50, 2.40	Glutathione metabolism	Amino acid
Proline	4.16, **3.33**, 3.32, 3.31, 2.08	Urea cycle; Arginine and Proline Metabolism	Amino acid
3‐Hydroxybutyrate	4.16, 2.40, **2.31**, 1.20	Ketone Bodies	Lipid
Lactate	**4.12**, 1.33	Glycolysis, Gluconeogenesis, and Pyruvate Metabolism	Carbohydrate
Myo‐inositol	4.07, **3.63**, 3.29	Inositol metabolism	Lipid
Choline	4.07, **3.21**	Glycerolipid metabolism	Lipid
Creatinine	**4.05**, 3.04	Creatine metabolism	Amino acid
Serine	4.04, **4.00**	Glycine, serine, and threonine metabolism	Amino acid
2‐hydroxobutyrate	4.04, 0.94, **0.93**	Cysteine, methionine, SAM, taurine metabolism	Amino acid
Creatine	3.93	Creatine metabolism	Amino acid
Valine	3.61, **1.04**, 0.99	Valine, leucine, and isoleucine metabolism	Amino acid
Glycerol	**3.58**, 3.66	Glycerolipid metabolism	Lipid
Glycine	3.56	Glycine, serine, and threonine metabolism	Amino acid
Proline	3.33	Urea cycle; Arginine and Proline Metabolism	Amino acid
Phosphocholine	**3.21**, 4.16	Phospholipid Metabolism	Lipid
Dimethyl sulfone	3.16		Gut microbiota
Ethanolamine	3.13	Glycerolipid metabolism	Lipid
Ornithine	3.06	Urea cycle; Arginine and Proline Metabolism	Amino acid
Albumin lysyl	2.99	Protein	Lipid
Asparagine	**2.86**, 2.83	Alanine and aspartate metabolism	Amino acid
Lipids = C‐CH_2_‐C=	2.75	Fatty Acid Metabolism	Lipid
Citrate	2.68, **2.54**	Krebs cycle	Energy
Glutamine	2.50, **2.45**, 2.14	Glutamate metabolism	Amino acid
Pyruvate	2.38	Glycolysis, Gluconeogenesis, and Pyruvate Metabolism	Carbohydrate
Glutamate	**2.36**, 2.08	Glutamate metabolism	Amino acid
Acetoacetate	2.27	Ketone bodies	Lipid
Adipic acid	**2.23**, 1.54	Fatty Acid, Dicarboxylate	Lipid
Glutathione	2.19	Glutathione Metabolism	Amino acid
Acetyl signals from glycoproteins	2.04	Glycoprotein	Lipid
Lipids CH_2_‐C = C	1.99	Fatty Acid Metabolism	Lipid
Acetate	1.92		Gut microbiota
Lysine	1.89, **1.71, 3.03**	Lysine metabolism	Amino acid
Arginine	1.89, **1.59**	Urea cycle; Arginine and Proline Metabolism	Amino acid
Alanine	1.48	Alanine and aspartate metabolism	Amino acid
Lipids CH_3_(CH_2_)*_n_*	1.26	Fatty Acid Metabolism	Lipid
Isopropanol	1.18		Gut microbiota
Isobutyrate	1.08		Gut microbiota
Isoleucine	**1.01**, 0.94	Leucine, Isoleucine, and Valine Metabolism	Amino acid
Leucine	0.96	Leucine, Isoleucine, and Valine Metabolism	Amino acid
3‐methyl‐oxovalerate	0.92	Leucine, Isoleucine, and Valine Metabolism	Amino acid
Lipids ‐CH_3_ (HDL)	0.84	Fatty Acid Metabolism	Lipid
Not identified integrated peaks	1.06, 1.15, 1.19, 1.36, 1.38, 1.42, 1.43, 1.44, 1.81, 2.06, 6.81		

Note

Metabolites are listed in descending order according to their resonant frequency in ppm. Numbers marked in bold indicate the frequency selected for analyses.

**Table A3:** Metabolites driving separation of ^1^H NMR metabolomic profiles between males and females determined by multivariate and univariate analyses

Metabolite	Pathway	Superpathway	Males vs. Females	
				*p*‐value
Fumarate	Krebs cycle	Energy	↑	.616
Glycerol	Glycerolipid metabolism	Lipid	↓	.321
Albumin lysyl	Protein	Lipid	↓	**.008**
Mannose	Fructose, Mannose and Galactose Metabolism	Carbohydrate	↓	.443
Creatinine	Creatine metabolism	Amino acid	↓	**.027**
1‐Methylhistidine	Histidine metabolism	Amino acid	↓	.540
Alanine	Alanine and aspartate metabolism	Amino acid	↓	.335
Lysine	Lysine metabolism	Amino acid	↓	.117
Glutamine	Glutamate metabolism	Amino acid	↓	.208
Phenylalanine	Phenylalanine & tyrosine metabolism	Amino acid	↓	.250

Note

Metabolites presented have l |w*|‐value ≥ 0.15 from the OPLS‐DA mode. *p*‐value based on a *t*‐test.

**Table A4:** Detailed characterization of two‐class OPLS‐DA models obtained based on ^1^H NMR metabolomic data distinguished by moose winter diet types

OPLS‐DA Model	Number of components^a^	Number of samples	R^2^X(cum)	R^2^Y(cum)	Q^2^(cum)	*p*‐value CV‐ANOVA
Generalist vs. *Pinus*	1 + 1	218	0.341	0.384	0.326	<.001
Saliseae vs. *Pinus*	1 + 1	201	0.285	0.478	0.348	<.001
*Betula* vs. *Pinus*	1 + 1	223	0.308	0.593	0.509	<.001
Saliceae vs. Generalist	1 + 1	37	0.355	0.601	0.334	.009
*Betula* vs. Generalist	1 + 1	59	0.413	0.664	0.568	<.001
*Betula* vs. Saliceae	1 + 1	42	0.266	0.697	0.449	<.001

^a^ The number of predictive components followed by the number of orthogonal model components.
